# Perspectives of municipalities on people living with incurable illness, palliative care and the end of life: A case study research

**DOI:** 10.1177/26323524261480745

**Published:** 2026-09-24

**Authors:** Trudy Schutter, Ian Koper, Marieke Groot, Kris Vissers, Jeroen Hasselaar

**Affiliations:** 1 Primary and Community Care, 6034 Radboud University Medical Center, Nijmegen, The Netherlands; 2 Agora, Bunnik, The Netherlands; 3 PZNL, Utrecht, The Netherlands; 4 6985 Rotterdam University of Applied Sciences, Rotterdam, The Netherlands; 5 Anaesthesiology, Pain and Palliative Medicine, 6034 Radboud University Medical Center, Nijmegen, The Netherlands

**Keywords:** palliative care, public health, public policy, social determinants of health, social participation, social work, social support system, supportive care

## Abstract

**Background:**

Increasing attention is being paid to community-based approaches to living with incurable illness, caregiving, dying, death, and bereavement. However, the role of local authorities and welfare organisations in addressing these themes remains unclear.

**Objectives:**

This study aims to explore the roles of Dutch municipalities and welfare organisations in supporting individuals living with incurable and life-limiting illness and their caregivers, and to examine the extent to which issues related to living with incurable illness, caregiving, dying, death, and bereavement ared addressed in local policy and practice.

**Design:**

A multiple case study was conducted across three cases in the Netherlands, each comprising a local authority (municipality), welfare organisation(s), and palliative care providers.

**Methods:**

Data were collected through semi-structured, in-depth interviews and analysis of local and regional policy documents and online information. In total, 27 professionals participated, including municipal civil servants, social professionals from welfare organisations, and palliative care professionals. Additionally, case specific policy documents were analysed.

**Results:**

Municipalities did not explicitly address palliative care and related end-of-life themes in policy and did not show designated responsibility. These topics were mainly embedded within broader ageing policy and addressed reactively. Social professionals provided support from a non-medical, person-centred perspective, focusing on everyday life and informal networks. Cross-domain collaboration occurred but varied between cases.

**Conclusion:**

Palliative care and related end-of-life themes are not structurally embedded within municipalities and welfare organisations but are addressed indirectly and to varying degrees at the local level. Greater municipal involvement, for example in raising societal awareness and increasing death literacy, could strengthen community-based palliative care.

## Introduction

In many Western countries, healthcare systems are increasingly challenged by demographic ageing, rising healthcare expenditures, and the growing prevalence of chronic and incurable diseases. These pressures have contributed to a policy shift towards addressing health and social care needs and challenges within communities and the welfare domain, rather than solely within the healthcare system, with municipalities taking on a increasingly prominent role.^1,2^ (See Appendix 1 for a glossary of relevant policy terms used in this article).

Concurrently, a conceptual shift has occurred within the field of palliative care, with greater emphasis on social and societal aspects of living with incurable and life-limiting illness, caregiving, death, dying and bereavement.^3^ Concepts like compassionate communities and public health palliative care highlight that care in the last phase of life is not solely a healthcare responsibility, but requires continuous involvement from citizens, informal networks, and local communities.^4,5^ This approach suggests an important role for societal actors beyond healthcare providers, including municipalities and welfare organizations.

However, empirical research on how municipalities and welfare organisations address palliative care and themes related to the last phase of life remains limited. The integration of these topics into their policies and practices is unclear, as is the nature of collaboration between municipalities, welfare organisations, informal networks, volunteers, and healthcare providers. Given the ongoing shift towards community-based approaches, addressing this knowledge gap is crucial for developing effective public policy.

This study explores the current and potential roles of municipalities and welfare organisations in addressing palliative care, incurable illness, related caregiving, death, dying, and/or bereavement. The research question guiding our study is: How and why do municipalities and welfare organisations incorporate or address palliative care, incurable illness, related caregiving, death, dying and/or bereavement in the support they provide to their residents? This concerns support provided to individuals who are no longer expected to recover, as well as to their loved ones, a phase that can last for months or even years,^2^ as well as initiatives aimed at societal awareness regarding the aforementioned themes.

## Methods

The SRQR guideline was followed for the reporting of this study.^6^

### Study design

This study employs a qualitative multiple case study approach, useful for explaining how or why a social phenomenon works.^7^ Three municipalities from the Netherlands were studied, each representing local practice and policy of the local authority and welfare organisation(s) regarding support for individuals and families facing end of life related themes; and initiatives to raise societal awareness about palliative care and related topics.

Central to each case were the perspectives of civil servants, municipal counsellors, and social professionals in local welfare organisations, obtained through semi-structured in-depth interviews. To broaden understanding, insights from local palliative care professionals and the regional palliative care network coordinator were also incorporated, alongside relevant case-specific policy documents. Additionally, local key informants identified by participants were approached and included. These individuals were referred to during one or more interviews and were considered by participants to possess specific knowledge, expertise, or experience relevant to the study topic.

Cases were selected based on accessibility, with variation between more rural and more urban municipalities. We hypothesized that city size could influence social cohesion and/or availability of services, which might influence the existence of social needs and appeals to the municipality and/or welfare organisation(s). Yet, we expected largely similar results across cases.

Participants received oral and written information, and provided informed consent. Ethical approval was sought from the Medical Ethical Committee East Netherlands (File number 2021-13359) and waived, as the study is not subject to the Medical Research Involving Human Subjects Act (WMO).

#### Inclusion criteria

Participants were eligible for inclusion when:

- Employed as a municipal government official and professionally involved in matters related to the study topic; or
- Employed as a social professional at a welfare organisation within the municipality and professionally involved in matters related to the study topic; or
- Employed as a palliative care network coordinator or palliative care nurse within the municipality; or
- Identified by other participants as key informant, with specific knowledge, expertise, or experience relevant to the study topic.

### Data collection

Potential participants were identified through online searches and snowball sampling and were approached by email or in person. Interested participants received an information letter and were given the opportunity to ask questions before providing written informed consent. Some individuals preferred informal conversations over recorded interviews (listed as “NA” in Table 1); insights from these conversations were collected with verbal consent. Data were collected from June 2024 to October 2025. Additional documentation provided by participants, was included in the analysis.

**Table 1.** Participants per case, along with their job function, and the organisation they are employed by.

Participant code	Job function	Organisation*
**Case A**		
A1	Neighbourhood manager	Municipality
A4	Municipal counsellor	Municipality
A6	Policy advisor Social support act	Municipality
NA	Neighbourhood mediation coordinator	Welfare organisation
NA	Community development worker	Welfare organisation
A2	Social worker	Welfare organisation
A5	Social work manager	Welfare organisation
NA	Community centre host	Community and welfare organisation
NA	Manager regional general practitioners organization	Regional GP organisation
A3*	Coordinator regional palliative care network	Regional palliative care network
**Case B**		
B1	Policy director welfare domain; former Social Support Act consultant	Municipality
B2	Policy director welfare domain	Municipality
B4	Social Support Act consultant	Municipality
B10*	Municipal counsellor	Municipality
B7	Community development worker; municipal council member	Welfare organisation
B5	Independent client support officer; Social worker; designated officer for elderly abuse; designated officer for informal caregiving	Independent client support organisation; Healthcare organisation
B3	Community nurse	Healthcare organisation
B6	Palliative care nurse, community nurse	Healthcare organisation
B8*	Coordinator regional palliative care network	Regional palliative care network
B9*	Program manager regional transformation plan for Advance Care Planning and palliative care	Independent professional
**Case C**		
C2	Neighbourhood manager	Municipality
C12*	Policy advisor	Municipality
NA	Social worker for the elderly	Welfare organisation
C6	Social worker for the elderly; former Social Support Act consultant	Welfare organisation
C7	Social worker for the elderly	Welfare organisation
C8	Social worker for the elderly (in training)	Welfare organisation
C9	Volunteer coordinator of a centre for life questions; university lecturer in spiritual caregiving	Welfare organisation
C4	Community development worker	Welfare organisation with a focus on community development
C10	Palliative care nurse	Healthcare organisation
C11	Palliative care nurse	Healthcare organisation
C3	Vice chair of a cancer support centre; retired General Practitioner	Local cancer support centre
C5	Coordinator regional palliative care network	Regional palliative care network

NA = Not applicable. Representing individuals who preferred to have an informal conversation, rather than a formal recorded interview. The insights from those conversations were permitted to be incorporated into the study.

*These participants provided additional information after the conducted interview. This information has been added to the analysis. The following documentation were included:

• A3: Personal email correspondence with local professionals about the subject matter;

• B10: Municipal vision document on the ‘Social Foundation’;

• B8/B9: Accepted regional transformation plan for Palliative Care and Advance Care Planning, and a specific memorandum outlining the interconnection of this plan with the welfare domain specifically;

• C12: Written preparation for the interview, which was coordinated with the policy team and the municipal counsellor.

**We distinguished three types of organisations:

• Municipalities: Municipal counsellors (aldermen) and civil servants (government employees) working for the municipality (local authority), at the town hall.

• Welfare organisation: Social professionals working at welfare organisations implementing the local welfare policy designed by the municipality

• Other organisations, such as the regional palliative care network 8 (appendix 1) and healthcare organisations.

The interviews were guided by an evolving topic list, reflecting emerging findings. The original topic list is presented in Textbox 1. Emerging findings from earlier interviews informed subsequent interviews, allowing topics of interest to be explored in greater depth with later participants. The interviews were audio-recorded, transcribed verbatim, and anonymised. The interviews lasted 30 to 67 minutes. Interviews were conducted by TS, who has a professional background in health policy sciences and spiritual caregiving and is experienced in conducting qualitative research interviews. TS knew participants C5 and C9 prior to the interview.

Most interviews were conducted at the participant’s workplace, some digitally via Teams, and one at the researcher’s workplace. Three interviews involved two participants, and one involved three. An overview is provided in table 1.

Initially, we searched for case-specific policy documents on the municipality’s or welfare organisation’s role in palliative care-related support. As none were identified, we expanded the search to broader relevant documentation. For each case we analysed:

- Welfare organisation’s website(s), and linked websites (snowball sampling)
- Municipality’s public website, and linked websites (snowball sampling)
- Local coalition agreement
- Regional strategy document(s), derived from national healthcare accords

**Textbox 1. Topic list interviews**

⁃ Introduction: Could you describe your role and position within your organisation and its broader context? What relationships and collaborations do you have with other stakeholders? Could you describe your previous professional experience and any other relevant roles or activities?
⁃ How would you describe the welfare domain?
⁃ In your work, are you involved with (policies concerning) people with incurable illnesses, people in the last years of life, and/or their relatives or bereaved family members?
⁃ What forms of social support are available to these individuals? Who provides this support? In what ways do you, if at all, support these individuals in your professional role? How is coordination or collaboration with other professionals and organisations organized?
⁃ To what extent do you consider municipalities to be responsible for supporting these individuals? (To what extent is this topic reflected in municipal policy?)
⁃ Municipalities are responsible for strengthening the “Social Foundation”. To what extent do you consider attention to serious illness, dying, death, and bereavement to fall within this responsibility? Why or why not? If not, whose responsibility do you consider it to be?
⁃ How do municipalities and welfare organisations collaborate on these issues? And how do they collaborate with other stakeholders?
⁃ Are there any other individuals you would recommend we interview?

### Data analysis

Interview and document data were analysed inductively using open and axial coding. TS performed the coding; codes were weekly discussed with IK and monthly with the full research team (KV, MG, JH, IK, TS). This group contained professionals with a medical, nursing, social and health science expertise in palliative care. JH and TS additionally reviewed the logbook and the development of themes. Atlas. ti was used to support the data management and -analysis.

Websites and linked pages were searched in June 2025 for publicly available local information and support related to end of life issues. Search terms were the Dutch equivalents of: death, palliative, end of life, hospice, incurable, terminal, grief, informal care, and family caregiving.

## Results

The cases differ in three respects: i) in local context (see Textbox 2), ii) in the professionals who agreed to participate (see Table 1), and iii) in the way each case addresses attention for themes related to the end of life. Participant inclusion was complex: the welfare domain is structured differently across cases, with varying job titles, and some invitees declined participation, citing insufficient knowledge. The results present cross-case findings and, when relevant, case-specific examples. Participant quotations are given to illustrate the findings.

**Textbox 2. Characteristics and local context of included cases in the Netherlands**

Case A represents a local practice in a municipality with a historic city status, home to around 50,000 residents. The social professionals and neighbourhood manager worked in an area with a high proportion of residents with a migration background and many single-person households. This specific neighbourhood uses social services more than the municipal average.

In case A, participants often referred to the Social Prescribing Program (in Dutch: *welzijn op recept*), which promotes collaboration between healthcare and welfare, and attention for (the cause of) psychosocial problems. Through this program, general practitioners (GPs) refer patients to a designated social professional for additional support^9,10^ (Appendix 1).

Case B represents a local practice in a municipality with a more rural profile than Case A. The municipality includes a city of about 30,000 residents, a village with 4,000 residents, and several small hamlets. Specific for Case B was an accepted Regional Transformation Plan for Palliative Care and Advance Care Planning (appendix 1).

In Case B, participants often referred to multidisciplinary, cross-domain team meetings involving both primary care and social professionals.

Case C represents a local practice in a municipality with a distinctly urban profile, including a historic university city of about 200,000 residents, considered medium to large by Dutch standards. Two welfare organizations were active in Case C. Additionally, two local community organisations were seen as relevant by several participants: a local Centre for Existential support, staffed by volunteers and a coordinator, embedded within one local welfare organisation, and the local Institution for Psychosocial Oncology (IPSO) centre, staffed by both volunteers and professionals (appendix 1).

In Case C, participants frequently referred to two programs promoting collaboration between healthcare and welfare: the Social Prescribing Program (*welzijn op recept*) and a local initiative focused on vulnerable elderly individuals with memory problems living at home.

### No explicit attention for palliative care and the end of life by the municipalities

The data reveal that palliative care, incurable illness, death, dying and bereavement are not specific policy focal points in any of the municipalities. No dedicated staff member is assigned to these themes, in policy or implementation. Municipalities appear to be exploring their approach; across cases this role is framed as related to strengthening the community-based social infrastructure (in Dutch: *Sociale basis*, in this article translated as “Social Foundation”) and fostering a caring society (appendix 1). The issues are considered relevant due to municipal responsibility for resident’s wellbeing, particularly in light of population ageing. Municipal websites mainly provide information on death registration and cemeteries, but not on palliative care, hospices, or other end-of-life services.

The “Social Foundation” is a policy term referring to the community-based social infrastructure (appendix 1):

*“The Social Foundation essentially refers to the network you have that enables you to lead a happy life. […] there is the personal, the communal, and the institutional Social Foundation”* (participant C2, neighbourhood manager).

While increased attention to the Social Foundation is expected to be beneficial, transformation will likely take years. Municipalities are still exploring their roles in palliative care and end-of-life themes, particularly because these topics are not specifically addressed within the Social Support Act. Its open character allows both more generous and more restrictive policy interpretation:

*“Health care insurers have a firm framework and do not deviate from it. But we are still… Also because the Social Support Act is a more flexible law, right? We don’t really have a clear boundary that you can draw*” (participant A6, policy advisor).

When addressed, palliative care and related themes are usually embedded within broader elderly support. Support related to the end of life for young(er) individuals, or parents with young children, is seen as exceptional and more urgent. In contrast to support in palliative care, municipalities’ role in dementia support, as well as dementia-friendly communities, are more clearly defined and established.

Although municipalities do not proactively focus on palliative care provision, they do become involved when appealed to. For example, in case C, when the organisation providing hospice services at the local hospice location informed the municipal counsellor that they planned to discontinue, the municipal counsellor engaged despite this not being a formal responsibility:

*“In the end, it became quite a significant issue within the municipality. A petition was launched, with residents expressing their desire for the hospice to remain operational. This led to council questions being raised. […] This also shows that the issue is alive, and as a municipality, in the end, you are doing it for your residents. Perhaps it’s not something we are directly responsible for, but you can see that it is a topic that resonates in the city”* (participant C12, policy advisor).

The data suggest that end-of-life topics are rarely addressed top-down, but that municipalities can support or facilitate initiatives that emerge from within the community. Still, the perceived level of responsibility varies across municipalities and among civil servants and counsellors, with personal experience and individual views influencing perspectives on the municipal role.

### Social professionals focus on individual or community needs, medical diagnosis or end of life does not influence their approach

Social professionals approach the palliative phase in a non-medical manner, often without formal training on the subject. Data shows that their role focuses more on strengthening and complementing the informal domain, than on extending healthcare professionals’ work. In all three cases, involvement typically occurred before the final stage of life or after death, rather than during the terminal phase, and often included support for informal caregivers. Social professionals in this study were either social workers, offering guidance, or community workers, promoting neighbourhood cohesion (appendix 1). While lacking specialized training, all encountered and addressed these issues, and responded through practical support, listening and human presence.

Social professionals’ approach differs from healthcare professionals’ focus on diagnosis and protocols. Instead, they respond to individual needs with tailored, person-centred support, sometimes simply by “being there”:

*“It is part of life, I would say. It is part of living here in the neighbourhood, meeting people who have their own struggles. […] And when people are terminally ill or about to pass away, than it is more… not that I specifically address that theme, it’s more about the person themselves, I would say”* (participant C4, community worker).

Their role appears to sustain everyday life, rather than extending or substituting for healthcare services:

*“[People without a diagnosis] or those with a diagnosis, such as diabetes or any other chronic illness they’ve been dealing with their entire lives, often end up in welfare services. They are relatively outside the scope of healthcare institutions and make extensive use of these services. […] I also think that social work largely reaches the lower-educated group, because people who are more financially well-off tend to easily go to more expensive services”* (participant C9, volunteer coordinator).

It appears that the involvement of social professionals often ends when individuals enter the formal healthcare system, for instance, when a case manager becomes involved.

Although incurable illness, death, dying and bereavement are not primary focal points for social professionals, respondents reported encountering and supporting individuals facing these issues. Rather than directly addressing death and dying, they typically assist with managing decline related to aging. In Case C, aging and bereavement are a specific focus of the welfare organisations, including grief support groups and proactive outreach to recently bereaved individuals:

*“In our municipality, as far as I know, everyone who turns 70, 75, 80, 85 receives a letter from our organisation to initially get in touch with a volunteer: to discuss what to expect as an older person, but also if a partner passes away, because we also conduct partner loss conversations”* (participant C7, social worker).

The local Centre for Existential support in Case C was also frequently mentioned as an important entity, offering support through volunteers who facilitate conversations, and discussions on mortality, and who occasionally assist with practical matters like drafting an advance directive or euthanasia declaration. The interview data suggests that GPs, spiritual counsellors, trained volunteers, and local churches are seen as the designated professionals or organisations for addressing themes related to the end of life. For some participants, this is linked to the personal character of these topics. Some social professionals felt insufficiently competent, and preferred referral of complex cases to healthcare professionals.

In Case A, social professionals consistently explored alternative support options, like volunteers or neighbours, while ensuring individuals received necessary support. This approach was supported by the municipality. While this may also apply to the other cases, this could not be confirmed in the study data. In Case B, the community worker noted that such an approach requires professional autonomy, sufficient time, municipal consensus, and adequate staffing.

### Collaboration can take various forms

All three cases showed examples of collaboration between social and primary care professionals, but these were without a specific focus on end-of-life themes. In Case C, palliative care nurses referred patients to the welfare domain and organized a symposium on quality of life to connect healthcare providers, social professionals, the municipality and the housing cooperation. Still, their day-to-day collaboration with the municipality appeared to be hindered by bureaucratic challenges:

*“I am always looking for the smartest way to get something done, because, for example, domestic assistance is just not longer available these days, even though it is urgently needed when someone is terminal. The municipality is a bureaucratic organisation where you can’t get through to arrange things quickly. Sometimes I manage to get it done, sometimes I don’t, and then they (the municipality) say later: ‘Why didn’t you mention it earlier? We could have done something sooner’”* (participant C11, palliative care nurse).

In Case B, collaboration with GPs, and with social professionals, was not self-evident for the participating palliative care nurse:

*“A lot is discussed about that in the training. Like, seek that collaboration. Brainstorm together. Involve other disciplines. I do find it difficult to find my position in that. You have general practitioners who are up to it, especially the younger generation. But you also have general practitioners who are really like, ‘What are you getting involved in? Why are you here? Why are you calling me to say someone is in pain? I was there myself yesterday’”* (participant B6, palliative care nurse).

At the organizational level, collaborations varied. In Case A, the palliative care network coordinator focused on aligning with municipalities, emphasizing their role in raising societal awareness and facilitating education on incurable illness, death, dying, bereavement, and palliative care:

*“Last year we began working on a regional transformation […]. In this process, we noticed that there is a significant difference [between municipalities] in mindset, a lot of variation in execution, ideas, and also in the level of involvement in the palliative care network. We’ve now agreed to focus primarily on societal awareness as a theme where the municipality is involved and engaged”* (participant A3, palliative care network coordinator).

In contrast, the network coordinator in Case B had little contact with municipalities but supported grassroots community initiatives such as art-based activities addressing mortality:

*“We often refer to the welfare domain as something institutionalized, while, when I look around, a lot is happening in the towns and villages themselves, where entire communities exist. And these communities are, well, I don’t know, caring for each other”* (participant C5, palliative care network coordinator).

The transition plan in Case B aimed to promote high-quality, proactive, transmural palliative care. Key aspects for the envisioned roles of the municipality and the welfare domain included: participating in cross-domain consultation, informal caregiver support, volunteer involvement, psychosocial and practical support, and societal awareness efforts. Training social professionals and involving municipalities were considered essential for successful transformation of palliative care.

## Discussion

We conducted a case study research in three municipalities, each involving at least ten individual participants and relevant documents and online information. Current Dutch healthcare agreements show policy attention for collaboration between the healthcare and welfare domain in palliative care. Yet, in our study we found that municipalities and welfare organisations not explicitly foster collaborations or prioritize end-of-life themes in policy and practice currently.

### The municipality should improve societal awareness

To establish connection between palliative care and the welfare domain at the local level, coordination between local authorities and the (coordinator of the) regional palliative care networks are essential. This can, as shown in this study, be achieved through joint development of policy plans, by integrating with each other’s consultation processes, or by jointly organising public events aimed at raising societal awareness. By working together and jointly shaping public awareness, an initial step is taken to move end-of-life issues and related themes from the healthcare domain to, or in collaboration with, the welfare domain. This requires careful consideration, as discussing ageing accompanied by decline, and human mortality, may be sensitive topics. Furthermore, municipalities are well-positioned to support and facilitate community-driven initiatives. This may include efforts to preserve a local hospice, as well as supporting volunteer organisations, art exhibitions, and other grassroots initiatives focusing on incurable illness, caregiving, death, dying and/or bereavement.^4,11^ This vision should be reflected in policy, which currently, at best, only mentions palliative care in relation to the elderly but often lacks concrete measures for collaboration between both domains concerning incurable illness, caregiving, death, dying, bereavement and/or palliative care.^11,12^ As stated by the Lancet Commission on the Value of Death, relationships are fundamental to death, dying and grief,^4^ therefore, municipal efforts to strengthen the Social Foundation are highly relevant.^13^

### Towards a community-approach to end of life issues

Social professionals, though typically not specialized or trained in supporting individuals living with incurable illness, death, dying, or bereavement, do encounter these issues in their work. The primary role of social professionals appeared to be offering support as needed, not guided or prioritized by medical diagnosis or phase of life, but in response to emotional or practical support questions.^14^ If collaboration between the healthcare and welfare domain in palliative care is envisaged, it is important to reflect on the role of social professionals in this process.

Most respondents framed end of life issues within the context of strengthening the Social Foundation and fostering a caring society. Participating palliative care professions, in contrast, emphasised that the welfare domain was largely unfamiliar to them, but could play a more prominent role in palliative care, with training of social professionals in palliative care considered important.^15^ Notably, both groups used different vocabularies, which might reflect a common limitation of palliative care: palliative care is often perceived as solely terminal care, leading to limited long-term collaboration with palliative care providers.^16^ Yet, an increasing number of individuals live for many years after being diagnosed with incurable illness, requiring support beyond the medical realm. Current palliative care organisation seem to hinder this broader approach of palliative care, starting from the diagnosis of incurable illness, which is necessary for improving quality of life during the remaining time.^16^ Strengthening collaboration with the welfare domain and social professionals could help meet this long-term needs.

To foster connection and alignment between palliative care and social professionals, familiarity with each other’s expertise, finding common vocabulary, and defining a clear division of roles without losing connection are required.^12,15^ For instance, we envision integrating end-of-life issues into the development of a caring society, and making concrete how incurable illness, related caregiving, dying, death and bereavement can be addressed within the Social Foundation.^17,18^ Also, this can involve raising awareness among social professionals about specificities of palliative care, and about the existence of volunteer organisations focusing explicitly on social support in the palliative phase.^15,19,20^

### Strengths and limitations

Case study research is sometimes criticized for alleged lack of rigour, possible bias of personal interpretation, and lack of generalizability.^21^ We ensured rigour by following the study protocol and logging any research activities. Also, in thoroughly describing our study design, data collection, and analysis, we aim to ensure transparency in our methodology, and reliability of our research. To counteract personal bias, the whole research team, with various backgrounds, engaged in monthly discussion and dialogue. Also, various bilateral consultations were conducted. Generalization in case study research refers not to sample representativeness, but to generalizing to (prior) theory.^7,22^ By thoroughly describing contexts, participants, and results, including quotations, we aimed to help readers determine if the findings are applicable to their own situation.^23^

Before data collection, we anticipated similar results across cases. We found differences in the presence of organisations and job functions, in collaboration opportunities and in participant’s’ personal beliefs. While results did not conflict, each case emphasized different aspects. Despite these variations, the outcomes appeared to reflect the same themes. As the themes were not systematically addressed, identifying key figures proved challenging, this was partly alleviated through the use of snowball sampling.

### Future research

Given the central role of the Social Foundation in the welfare domain's policy, it would be valuable to systematically study the perspectives of families, communities, and the dynamics between the welfare domain and healthcare domain in palliative care.^24^ This could enhance our understanding of our societal death system, which is the “interpersonal, socio-physical, and symbolic network through which an individual’s relationship to mortality is mediated by society”,^25^ enriching discussions on the role of municipalities and welfare organisations in these areas. Also, specific research into what social and palliative care professionals need in order to fulfil their role in a community-based approach to incurable illness, caregiving, death, dying, and bereavement, could help strengthening collaboration between those domains.

## Conclusions

Palliative care and related end-of-life themes are not structurally embedded within (Dutch) municipalities and welfare organisations, but are addressed indirectly and to varying degrees at the local level. Municipal involvement, for example in societal awareness around these themes, by increasing death literacy, and by promoting a caring society, could help shape community-based palliative care. This also requires engagement between social professionals and palliative care professionals.

## Data Availability

The data generated and analysed during the current study are available from the corresponding author on reasonable request.*

## Appendix

**Appendix 1.** Glossary

Caring society	The ambition for the “Social Foundation” is a resilient, inclusive, and caring society in which people can thrive, look out for one another, and contribute to their own and others wellbeing.^26^ There is no distinct definition, but the term is widely used in the Netherlands, along with similar terms such as caring neighbourhood and caring community.
IPSO centre	*“In the Netherlands, Institutions for Psychosocial Oncology (IPSO) centres provide easily accessible psychosocial support to individuals affected by cancer. There are [100] IPSO centres in the Netherlands, each having at least one paid coordinator/manager and several trained volunteers”*.^27,28^
Regional Palliative Care Networks	*“There are 65 regional palliative care networks in the Netherlands, financed by the Dutch Ministry of Health, Welfare and Sport, to support interdisciplinary integrated palliative care. These regional networks are formal and sustainable partnerships of independent organisations that are involved with palliative care in a specific geographical region. Each network has a network coordinator who is the primary liaison for communication and coordination between the different formal healthcare providers and informal support organisations. The network coordinator supports the development of the network and improvement of the quality of care within the region of the network and has an overview of palliative care (services) in the region”*.^8,29^ The network coordinator does not provide individual care or support herself.
Regional Palliative Care Transformation plan	To improve the healthcare in the Netherlands and to prepare it for the future, there are national health care agreements.^30^ One of the topics addressed in these agreements is collaboration between the various domains of care and support at the regional level.
	All regions have a regional strategy document, the three cases central in this study are all situated in another region, the strategy documents of these three regions were included in the study.
	These three regions have also submitted a palliative care transformation plan, aiming to make palliative care part of the regular care process. The region of Case B, unlike both other regions, received finances to put these plans into practice. This plan is added to the analysis. The other two regional transformation plans were not available for inclusion.
Social Foundation (sociale basis)	A Dutch policy term about the community-based social infrastructure that is widely used today, often without a distinct definition. We follow the definition given by Movisie & Verwey-Jonker Instituut^26^ that was also used by participants:
	The Social Foundation encompasses three spheres: the personal, communal and institutional Social Foundation:
	• Personal: an individual’s personal network
	• Communal: Communities, which can be formal networks such as associations, or informal groups such as a walking group. The communal Social Foundation also includes occasional and structural volunteering.
	• Institutional: Facilities, services and paid professionals. This includes community centres, libraries, and health centres; collective services, volunteer support points, informal caregiver support, volunteer organizations, etc.; and social workers, teachers, housing consultants from housing corporations, community nurses, neighbourhood police officers, et cetera.
Social Prescribing Program (Welzijn op Recept)	*“‘Welzijn op Recept’ is a programme that allows primary care providers to refer patients with psychosocial problems to a local social well-being organisation. […] Welzijn op Recept seeks to offer support in maintaining and improving the health and well-being of people with psychosocial problems that do not require medical or psychological treatment. The objective is to enhance the participants’ quality of life by helping them re-engage in activities and create new social contacts”*.^9^
Social professional	Social professionals included in this study were either social workers of community workers. Social workers are typically focused on the provision of practical and emotional support at the individual or family level.
	Community workers are more focused on shaping a caring society and providing opportunities for meeting others through various social activities.
Social Support Act (Wmo 2015)	Municipalities are responsible for supporting individuals who are not self-sufficient to live at home for as long as possible. The municipality provides support at home through the Social Support Act (in Dutch: *Wmo 2015*). This includes for example guidance, day care services, and informal caregiving support.^31^
Welfare domain (Sociaal domein)	The welfare domain refers to the mandate municipalities have under the Social Support Act, the Participation Act, and the Youth Act. It encompasses the efforts of municipalities and societal organisations, including welfare organisations, to promote health, welfare, and safety for residents. This includes supporting residents, increasing the liveability in society, and fostering participation.^32^ The term 'welfare domain' may also be understood as referring to the municipal social care sector.
